# Validity of Medical Record Abstraction and Electronic Health Record–Generated Reports to Assess Performance on Cardiovascular Quality Measures in Primary Care

**DOI:** 10.1001/jamanetworkopen.2020.9411

**Published:** 2020-07-28

**Authors:** Juell Homco, Hélène Carabin, Zsolt Nagykaldi, Tabitha Garwe, F. Daniel Duffy, David Kendrick, Sydney Martinez, Yan Daniel Zhao, Julie Stoner

**Affiliations:** 1 Department of Medical Informatics, School of Community Medicine, University of Oklahoma Health Sciences Center, Tulsa; 2 Department of Biostatistics and Epidemiology, University of Oklahoma Health Sciences Center, Oklahoma City; 3 Département de Pathologie et Microbiologie, Université de Montréal, Montréal, Quebec, Canada; 4 Département de Médecine Sociale et Préventive, Université de Montréal, Montréal, Quebec, Canada; 5 Centre de Recherche en Santé Publique, Université de Montréal et Centre Intégré Universitaire de Santé et de Services Sociaux du Centre-Sud-de-l’Île-de-Montréal, Montréal, Quebec, Canada; 6 Department of Family and Preventive Medicine, University of Oklahoma Health Sciences Center, Oklahoma City

## Abstract

**Importance:**

Cardiovascular disease is the leading cause of death in the United States. To improve cardiovascular outcomes, primary care must have valid methods of assessing performance on cardiovascular clinical quality measures, including aspirin use (aspirin measure), blood pressure control (BP measure), and smoking cessation counseling and intervention (smoking measure).

**Objective:**

To compare observed performance scores measured using 2 imperfect reference standard data sources (medical record abstraction [MRA] and electronic health record [EHR]–generated reports) with misclassification-adjusted performance scores obtained using bayesian latent class analysis.

**Design, Setting, and Participants:**

This cross-sectional study used a subset of the 2016 aspirin, BP, and smoking performance data from the Healthy Hearts for Oklahoma Project. Each clinical quality measure was calculated for a subset of a practice’s patient population who can benefit from recommended care (ie, the eligible population). A random sample of 380 eligible patients were included for the aspirin measure; 126, for the BP measure; and 115, for the smoking measure. Data were collected from 21 primary care practices belonging to a single large health care system from January 1 to December 31, 2018, and analyzed from February 21 to April 17, 2019.

**Main Outcomes and Measures:**

The main outcomes include performance scores for the aspirin, BP, and smoking measures using imperfect MRA and EHRs and estimated through bayesian latent class models.

**Results:**

A total of 621 eligible patients were included in the analysis. Based on MRA and EHR data, observed aspirin performance scores were 76.0% (95% bayesian credible interval [BCI], 71.5%-80.1%) and 74.9% (95% BCI, 70.4%-79.1%), respectively; observed BP performance scores, 80.6% (95% BCI, 73.2%-86.9%) and 75.1% (95% BCI, 67.2%-82.1%), respectively; and observed smoking performance scores, 85.7% (95% BCI, 78.6%-91.2%) and 75.4% (95% BCI, 67.0%-82.6%), respectively. Misclassification-adjusted estimates were 74.9% (95% BCI, 70.5%-79.1%) for the aspirin performance score, 75.0% (95% BCI, 66.6%-82.5%) for the BP performance score, and 83.0% (95% BCI, 74.4%-89.8%) for the smoking performance score.

**Conclusions and Relevance:**

Ensuring valid performance measurement is critical for value-based payment models and quality improvement activities in primary care. This study found that extracting information for the same individuals using different data sources generated different performance score estimates. Further research is required to identify the sources of these differences.

## Key Points

**Question:**

Do medical record abstraction and electronic health record–generated reports provide valid methods of determining estimates of performance of common cardiovascular care?

**Findings:**

In this cross-sectional study of a random sample of 621 patients eligible for recommended care, medical record abstraction resulted in higher performance scores compared with electronic health record–generated reports. However, misclassification-adjusted performance scores were more similar to electronic health record–generated performance scores in some cases.

**Meaning:**

These findings suggest that meeting a performance target may depend on the method used to estimate performance, which has implications for quality improvement efforts and value-based payment models.

## Teaser

This cross-sectional study compares observed performance scores measured using medical record abstraction and electronic health record–generated reports with misclassification-adjusted performance scores obtained using bayesian latent class analysis among patients treated in primary care practices.

## Introduction

Cardiovascular disease, which includes hypertension, coronary heart disease, heart failure, and stroke, is the leading cause of death in the United States. Each year, an estimated 935 000 individuals have a new or recurrent coronary event, defined as a myocardial infarction resulting in hospitalization or a coronary heart disease death.^1^ This does not include the additional 155 000 first-time myocardial infarctions that are thought to go undetected each year.^1^ The risk factors for cardiovascular disease are multifactorial, and therefore improving cardiovascular health will require multilevel interventions targeting individuals, health care practices, and communities.^2,3,4^

In 2011, the US Department of Health and Human Services created the Million Hearts campaign to promote partnerships among communities, clinicians, health systems, nonprofit organizations, federal agencies, and private-sector organizations to improve cardiovascular outcomes in the United States and achieve a common goal: prevent 1 million cardiovascular events by 2022.^5^ The program aims to improve clinical care by focusing on the ABCS of heart health. The ABCS are commonly reported cardiovascular clinical quality measures in adult primary care, reported as the proportion of patients receiving recommended care.^6^ These measures are consistent with US Preventive Services Task Force recommendations and encourage clinicians to recommend aspirin therapy for those at risk for cardiovascular events (aspirin), to control high blood pressure (BP), to manage cholesterol levels (cholesterol), and to provide smoking cessation intervention to smokers (smoking).^4,5,7,8,9^

Primary care practices usually rely on medical record abstraction (MRA) or electronic health record (EHR)–generated reports to determine their ABCS performance scores. Valid performance scores are critical for implementing successful quality improvement initiatives in practice. In addition, these measures are frequently reported in value-based payment models. Unfortunately, neither MRA nor EHRs are perfect data sources for determining whether performance is met or not. For example, a recommendation for aspirin use in a notes field would be missed in EHR-generated reports. Similarly, we found that aspirin could be documented 72 different ways on the medication list in 1 EHR system used by a single health system. Owing to human error, one of these may be missed by a medical record abstractor. These errors in correctly classifying patients as meeting performance introduce misclassification bias. Therefore, whether a patient received recommended care (performance met or not) is a latent variable, because it cannot be directly observed. Identifying and adjusting for misclassification bias, or error, is not a new epidemiological challenge and is investigated extensively in diagnostic accuracy studies by comparing each person’s true disease status with the value obtained from the test of interest.^10^ Using data from a large dissemination and implementation research project, this study aimed to apply the framework used in diagnostic accuracy studies to (1) compare observed performance scores measured using MRA and EHR-generated reports with misclassification-adjusted performance scores obtained using bayesian latent class analysis and (2) to compare the probability of meeting an 80% performance target for each performance score based on MRA, EHR, and misclassification-adjusted performance score estimates.

## Methods

This study was embedded into the Healthy Hearts for Oklahoma (H2O) Project, funded through the Agency for Healthcare Research and Quality EvidenceNOW: Advancing Heart Health in Primary Care, a stepped-wedge randomized trial aiming to improve ABCS performance scores to at least 80% in small- and medium-sized primary care practices across the state.^11^ This project was reviewed and approved by the University of Oklahoma Health Sciences Center institutional review board. A waiver of informed consent was granted by the institutional review board given that data analyses would be conducted using deidentified data. We adhered to the Standards for Reporting of Diagnostic Accuracy Studies That Use Bayesian Latent Class Models (STARD-BLCM) guidelines for the reporting of this study.^12^

### Definition and Measurement of Aspirin, BP, and Smoking Performance

The current cholesterol performance measure was not available from practice EHRs and therefore was not included in this study. The performance measures were extracted based on the following measure specification definitions: Centers for Medicare & Medicaid Services (CMS) 164v5.2, Ischemic Vascular Disease (IVD): Use of Aspirin or Another Antithrombotic; CMS 165v5, Controlling High Blood Pressure; and CMS 138v5, Preventive Care and Screening: Tobacco Use: Screening and Cessation Intervention.^13^ The MRA protocols and data extraction tools (eMethods 1 in the Supplement) used for the H2O Project were modified for this study.

### Data Sources and Study Population

This is a secondary analysis of data collected as part of the H2O Project. Bayesian sample size methods described by Dendukuri et al^14^ were used to determine the precision of estimates to be expected with a range of medical records to be abstracted for each performance measure using the PropMisclassSampleSize software. Details are provided in eMethods 2 and eTables 1 to 5 in the Supplement.

After a discussion with H2O leadership, it was determined that the project had resources to conduct 500 MRAs for the aspirin measure, 150 for the BP measure, and 150 for the smoking measure. Eligible patients seeking care during calendar year 2016 at 28 of the 263 primary care practices (10.6%) enrolled in the H2O Project initially willing and able to participate in MRA were sampled at random. Unfortunately, owing to unforeseen barriers, medical record abstractors were not able to gain access to 1 health care system for MRA. Therefore, MRA was conducted only among randomly selected eligible patients at 21 practices representing 1 large health care system using a single EHR vendor from January 1 to December 31, 2018. A total of 380 EHRs and medical records from 6654 eligible patients were abstracted for the aspirin measure; 126 from 28 428 eligible patients, for the BP measure; and 115 from 56 522 eligible patients, for the smoking measure. These sample sizes correspond to 95% bayesian credible interval (BCI) widths from 0.2 to 0.3 for the 3 performance measures, according to sample size calculations using the average length criterion (eMethods 2 and eTables 1-5 in the Supplement). Patient flow diagrams outlining how patients were identified and ultimately included in the final analysis for each performance measure are provided in eFigures 1 to 3 in the Supplement.

### Statistical Analysis

Data were analyzed from February 21 to April 17, 2019. The medians and 95% BCIs of the posterior distribution of the aspirin, BP, and smoking performance scores using the MRA and EHR data sources in combination with a diffuse prior (β [1.0, 1.0]) were used to represent the observed (ie, unadjusted) estimates. Classification contingency tables were constructed to visualize patient-level agreement between MRA and EHR classification for each performance measure.

Bayesian latent class models that assume that neither data source is a perfect reference standard were developed to estimate the sensitivity and specificity of MRA and EHR in classifying whether the performance for aspirin, BP, and smoking was met.^15,16,17^ More specifically, bayesian latent class models for 1 population and 2 imperfect tests were used, assuming and not assuming conditional independence. These models simultaneously use patient-level MRA and EHR data in combination with prior information to estimate the posterior distribution of misclassification-adjusted performance. Models taking conditional dependence into account resulted in very similar results and, therefore, only models assuming conditional independence are presented herein. Modeling did not account for clustering of patients within practices because population estimates of the true performance scores, summarizing data across all 21 practices, were not derived. The general framework for these models is provided in eMethods 3 in the Supplement.

**Figure 1.**
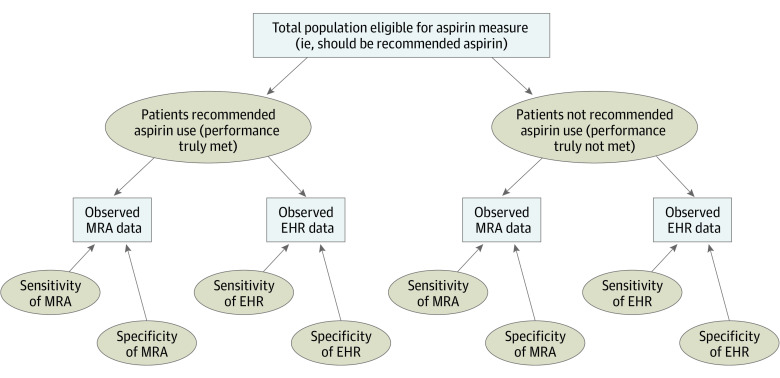
Schematic Illustration of a Bayesian Latent Class Model for the Aspirin Performance Measure Using Medical Record Abstraction (MRA) and Electronic Health Record (EHR)–Generated Data Rectangles represent observed data; ovals represent unknown (ie, unobserved) parameters.

Figure 1 illustrates the bayesian latent class models, using aspirin as an example, where rectangles represent observed data and dashed ovals represent unknown (ie, unobserved) parameters.^17,18^ All individuals eligible for the aspirin measure have either been truly recommended aspirin use or not (ie, performance was met or not). However, this is a latent variable because it is not directly known or observed. Medical record abstraction and EHR data were available for each individual in this analysis. Therefore, whether each individual is classified as having been recommended aspirin use or not by each of these data sources was observed and available. However, what was observed depended on the sensitivity and specificity of the MRA and EHR.

The results of each bayesian latent class model include a misclassification-adjusted median performance score estimate from the posterior distribution with 95% BCIs. Results also include a median MRA and EHR sensitivity and specificity with 95% BCIs. In addition, the proportion of the posterior density distribution above the threshold score of 80% meeting performance for aspirin, BP, and smoking was calculated for the adjusted and observed models. All analyses were conducted using WinBUGS software, version 1.4.3 (MRC [Medical Research Council] Biostatistics Unit, Cambridge University).

#### Prior Distributions

Two sets of priors were used in the bayesian latent class models. Prior set 1 was based on estimates of the sensitivity and specificity of EHR data to correctly classify patients as having performance met or not obtained from 2 subject-matter expert physicians (F.D.D. and D.K.) using the MATCH Tool.^19^ The same 2 experts were asked their professional opinion to determine priors for the MRA sensitivity and specificity for each performance measure. Prior set 2 used diffuse uniform distributions in the range of 0.5 to 1.0 for all parameters.

#### Sensitivity Analysis

A series of sensitivity analyses were conducted to explore alternative modeling choices. The robustness of models was tested by trying various sets of prior information, including relaxing priors as seen in prior set 2.

#### Model Fit and Convergence

Posterior estimates for each parameter of interest were generated using the Markov chain Monte Carlo sampling method and the Gibbs algorithm. Three simulation chains of 100 000 iterations were run with different initial values. The first 5000 iterations were discarded as the burn-in period. Convergence of the chains after the initial burn-in period was assessed by visually inspecting the history and density plots and the Brooks-Gelman-Rubin diagnostic plots.^20,21^

#### Bias

Bias was quantified as the difference between the observed posterior distribution of the performance scores according to the MRA and EHR and the posterior distribution of the misclassification-adjusted performance scores (ie, observed − adjusted). In addition, the relative percentage bias was calculated as [(observed – adjusted)/adjusted] × 100. Bias and relative percent bias were calculated using the bayesian latent class model results based on prior set 1.

## Results

### Observed Estimates of Aspirin, BP, and Smoking Performance Scores

**Figure 2.**
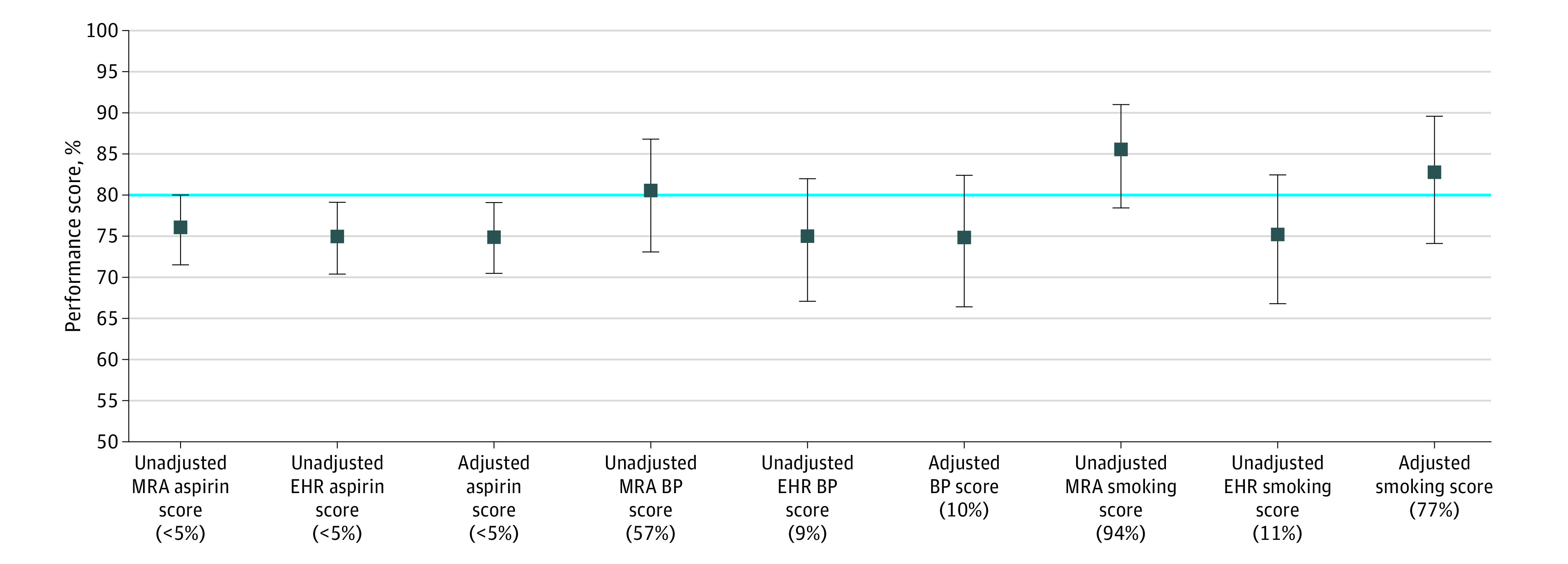
Observed Medical Record Abstraction (MRA) and Electronic Health Record (EHR)–Generated Scores and Prior Set 1 Results Results are based on expert opinion, misclassification-adjusted median scores for the aspirin, blood pressure (BP), and smoking performance measures. Percentages in parentheses represent the probability of meeting the 80% target. Error bars indicate 95% bayesian credible intervals.

A total of 621 eligible patients were included in the analysis. Table 1 shows the cross-tabulated results and the observed median performance scores and 95% BCIs for the aspirin, BP, and smoking measures. Medical record abstraction resulted in a larger number of patients with performance met, and therefore observed prevalence, for all 3 performance measures. Based on MRA and EHR data, observed aspirin performance scores were 76.0% (95% BCI, 71.5%-80.1%) and 74.9% (95% BCI, 70.4%-79.1%), respectively; observed BP performance scores, 80.6% (95% BCI, 73.2%-86.9%) and 75.1% (95% BCI, 67.2%-82.1%), respectively; and observed smoking performance scores, 85.7% (95% BCI, 78.6%-91.2%) and 75.4% (95% BCI, 67.0%-82.6%), respectively. Based on posterior density distributions, the probability of meeting the 80% target based on observed MRA and EHR data was 3% and 1%, respectively, for the aspirin measure; 57% and 9%, respectively, for the BP measure; and 94% and 11%, respectively, for the smoking measure (Figure 2).

**Table 1.** Classification Contingency Table of Whether Performance Was Met^a^

Performance parameter	Performance met according to MRA		
	Aspirin, No. yes/no	BP, No. yes/no	Smoking, No. yes/no
Performance met according to EHR^b^			
Yes	283/2	93/2	84/3
No	6/89	9/22	15/13
Observed median performance score, % (95% BCI)^c^			
MRA	76.0 (71.5-80.1)	80.6 (73.2-86.9)	85.7 (78.6-91.2)
EHR	74.9 (70.4-79.1)	75.1 (67.2-82.1)	75.4 (67.0-82.6)

Abbreviations: BCI, bayesian credible interval; BP, blood pressure; EHR, electronic health record; MRA, medical record abstraction.

^a^ Data were according to observed MRA and EHR.

^b^ Expressed as the number of patients for each performance met/not met.

^c^ The observed performance scores were obtained from the posterior distribution resulting from the combination of the likelihood obtained from either the MRA or EHR data with a diffuse prior.

### Misclassification-Adjusted Estimates of Aspirin, BP, and Smoking Performance Scores

All bayesian latent class models converged. Priors and posterior distributions for performance scores, sensitivities, and specificities for each model are summarized in Table 2. Misclassification-adjusted estimates were 74.9% (95% BCI, 70.5%-79.1%) for the aspirin performance score, 75.0% (95% BCI, 66.6%-82.5%) for the BP performance score, and 83.0% (95% BCI, 74.4%-89.8%) for the smoking performance score in prior set 1. Similar performance scores, sensitivities, and specificities were observed for prior sets 1 (based on expert opinion) and 2 (based on diffuse priors), although posterior estimates were more uncertain with prior set 2. Medical record abstraction posterior sensitivities were consistently above 95% across both prior sets (99.6% for aspirin, 98.2% for BP, and 97.7% for smoking prior set 1 and 99.4% for aspirin, 98.2% for BP, and 97.4% for smoking for prior set 2), and EHR posterior sensitivities were all nearly 90% or higher (96.8% for aspirin, 96.0% for BP, and 89.6% for smoking for prior set 1 and 98.8% for aspirin, 94.6% for BP, and 88.7% for smoking for prior set 2). There was notable uncertainty in the estimate for MRA specificity for BP across prior sets 1 (median performance score, 78.4% [95% BCI, 58.3%-97.7%]) and 2 (median performance score, 82.2% [95% BCI, 59.2%-99.0%]) and MRA specificity for smoking according to prior set 2 (median performance score, 70.7% [95% BCI, 51.0%-98.2%]). Otherwise, MRA specificity was greater than 95% for aspirin across prior sets (98.2% [95% BCI, 92.4%-99.9%] for prior set 1 and 95.8% [95% BCI, 89.1%-99.8%] for prior set 2) and was 86.0% (95% BCI, 80.2%-98.7%) for smoking in prior set 1. Electronic health record specificities were around 90% or higher for each performance measure across both prior sets (95.8% for aspirin, 94.9% for BP, and 89.2% for smoking for prior set 1 and 98.2% for aspirin, 93.2% for BP, and 86.8% for smoking for prior set 2). Overall, MRA was found to be more sensitive than EHR for each of the performance measures, and EHR was almost always more specific than MRA.

**Table 2.** Misclassification-Adjusted Aspirin, BP, and Smoking Performance Scores and Sensitivities and Specificities^a^

Variable	Misclassification-adjusted median performance score, % (95% BCI)		Prior distributions used in bayesian latent class models	
	Prior set 1^b^	Prior set 2^c^	Prior set 1^b^	Prior set 2^c^
**Aspirin**				
Performance score	74.9 (70.5-79.1)	75.4 (70.8-79.7)	β (13.59, 9.73); normal (0.58, 0.01)	Uniform (0.5, 1.0)
MRA				
Sensitivity^d^	99.6 (98.2-100.0)	99.4 (97.9-100.0)	Uniform (0.5, 1.0)	Uniform (0.5, 1.0)
Specificity^d^	98.2 (92.4-99.9)	95.8 (89.1-99.8)	Uniform (0.8, 1.0)	Uniform (0.5, 1.0)
EHR				
Sensitivity^d^	96.8 (94.2-98.5)	98.8 (96.3-99.9)	β (9.57, 5.18); normal (0.65, 0.01)	Uniform (0.5, 1.0)
Specificity^d^	95.8 (93.9. 97.3)	98.2 (93.4-99.9)	β (417.49, 20.78); normal (0.95, <0.01)	Uniform (0.5, 1.0)
**BP control**				
Prevalence	75.0 (66.6-82.5)	77.9 (68.1-85.9)	β (14.71, 8.99); normal (0.62, 0.01)	Uniform (0.5, 1.0)
MRA				
Sensitivity^d^	98.2 (93.6-99.1)	98.2 (93.6-99.9)	Uniform (0.5, 1.0)	Uniform (0.5, 1.0)
Specificity^d^	78.4 (58.3-97.7)	82.2 (59.2-99.0)	Uniform (0.8, 1.0)	Uniform (0.5, 1.0)
EHR				
Sensitivity^d^	96.0 (88.8-99.6)	94.6 (86.6-99.7)	β (42.06, 2.77); normal (0.94, <0.01)	Uniform (0.5, 1.0)
Specificity^d^	94.9 (85.8-99.2)	93.2 (76.6-99.7)	β (43.76, 5.75)	Uniform (0.5, 1.0)
			Normal (0.88, <0.01)	
**Smoking cessation counseling and intervention**				
Prevalence	83.0 (74.4-89.8)	82.3 (69.1-91.2)	β (11.16, 5.03); normal (0.69, 0.01)	Uniform (0.5, 1.0)
MRA				
Sensitivity^d^	97.7 (92.3-99.9)	97.4 (91.6-99.9)	Uniform (0.5, 1.0)	Uniform (0.5, 1.0)
Specificity^d^	86.0 (80.2-98.7)	70.7 (51.0-98.2)	Uniform (0.8, 1.0)	Uniform (0.5, 1.0)
EHR				
Sensitivity^d^	89.6 (83.4-94.9)	88.7 (78.8-98.5)	β (42.06, 2.77); normal (0.94, <0.01)	Uniform (0.5, 1.0)
Specificity^d^	89.2 (79.5-95.6)	86.8 (61.8-99.3)	β (43.76, 5.75); normal (0.88, <0.01)	Uniform (0.5, 1.0)

Abbreviations: BCI, bayesian credible interval; BP, blood pressure; EHR, electronic health record; MRA, medical record abstraction.

^a^ Prior distributions used in bayesian latent class models are provided. Equivalent normal (μ, σ^2^) distributions are presented for the beta (α, β) distributions.

^b^ Prior set 1 is based on prior elicitation from experts.

^c^ Prior set 2 represents diffuse priors.

^d^ Indicates with respect to the true (unknown) status of whether a patient received recommended care (ie, performance met or not).

Performance score results based on prior set 1 are illustrated in Figure 2 with observed estimates for comparison. Aspirin performance scores were very similar across all 3 data sources (observed MRA, observed EHR, and misclassification adjusted), although the misclassification-adjusted results were most similar to those obtained using observed EHR data. Based on posterior probability distributions, the probability of meeting the 80% target for aspirin was 1% according to the adjusted results. Misclassification-adjusted BP performance was also most similar to observed EHR performance and resulted in a probability of meeting the 80% target of 10%. Adjusted smoking performance was more similar to observed MRA results than EHR results. The probability of meeting the 80% target for smoking was 77% according to the adjusted estimates.

### Sensitivity Analysis

In general, the results were robust to different prior distributions, especially performance score and sensitivity estimates, which remained relatively precise across multiple models. However, this was not always the case with specificity estimates. The use of diffuse priors resulted in notable uncertainty in MRA specificities for smoking (70.7%; 95% BCI, 51.0%-98.2%).

### Bias

The bias and relative percentage bias estimates for aspirin, BP, and smoking performance score estimates are provided in Table 3. All but 2 measures resulted in estimates of relative bias of less than 5%, and all the 95% BCIs contained 0. However, BP measured with MRA overestimated the adjusted value by 5.63% (95% BCI, −4.89 to 16.17), and smoking measured with EHR underestimated the adjusted value by −7.62% (95% BCI, −18.43% to 3.59%).

**Table 3.** Median Bias and Relative Percentage Bias Introduced by MRA and EHR Data in Estimating Aspirin, BP, and Smoking Performance Scores

Data	Estimate (95% BCI), %		
	Aspirin	BP	Smoking
MRA			
Bias	1.04 (–5.01 to 7.13)	5.63 (–4.89 to 16.17)	2.64 (–7.19 to 12.87)
Relative percentage bias	1.39 (–6.45 to 9.95)	7.05 (–6.10 to 23.80)	3.17 (–8.20 to 17.01)
EHR			
Bias	0.02 (–6.10 to 6.14)	0.16 (–10.79 to 11.09)	–7.62 (–18.43 to 3.59)
Relative percentage bias	0.02 (–7.86 to 8.57)	0.22 (–13.48 to 16.26)	–9.16 (–21.17 to 4.70)

Abbreviations: BCI, bayesian credible interval; BP, blood pressure; EHR, electronic health record; MRA, medical record abstraction.

## Discussion

Despite the routine use of performance measurement in primary care, very little has been published on the validity of MRA and EHR data for this activity. Herein, we used a bayesian latent class approach to estimate the sensitivities and specificities of MRA and EHR data in the absence of a perfect reference standard and misclassification-adjusted performance scores. Our study is unique in that patient-level data were available for 21 practices, representing 1 large health care system. Bayesian latent class models are traditionally used to assess the validity of medical diagnostic tests, and their application to this research topic is extremely novel.^22,23^

These results raise significant concerns about the comparability and validity of different methods used to determine performance scores, which has important implications for quality improvement activities and implementation of value-based payment models. Despite being imperfect, our study illustrates that MRA and EHR data do a relatively good job of determining performance scores, given that there are no alternatives. However, using imperfect performance measures in practice can have significant implications when payment to clinicians depends on the achievement of target performance levels. The H2O Project aimed to improve ABCS performance scores to 80%. Although all data sources, adjusted or not, suggested that the aspirin performance target was consistently not met, the conclusions were different for BP and smoking. Indeed, the performance target would have been met for BP and smoking according to the MRA but not the EHR data. The misclassification-adjusted estimate resulted in performance met for smoking but not met for BP. When looking at how much of the posterior probability exceeded the 80% target, smoking performance would have been considered mostly met using MRA (ie, 94% above the target), but the conclusion would have been entirely different when using EHR data (ie, 11% above the target), with the adjusted estimate falling in between (ie, 77% above the target). Similarly, the 80% BP target would have been considered met with a probability of almost 60% using MRA but approximately 10% using EHR or the adjusted estimates. On the basis of these results, those administering payment could make practice payment tier based and adjust for the probability of meeting performance targets after misclassification adjustment.

### Limitations

Despite the novelty of this analysis, this project has limitations and caveats that should be considered when interpreting the results. One drawback of applying bayesian statistics to the relatively new field of performance measurement in health care is the limited amount of prior information available on their validity. However, this limitation was partly addressed by conducting a prior elicitation exercise that allowed the inclusion of expert knowledge in these analyses. Moreover, a variety of priors were evaluated, including poorly informative ones. Performance scores and sensitivity estimates were relatively similar across models using elicited vs diffuse priors, yet the results based on expert belief were generally more precise. On the other hand, specificity estimates were uncertain owing to limited prior information and the high-performance scores, especially for BP and smoking.

Medical record abstraction was not conducted for all patients deemed eligible for the aspirin, BP, and smoking measures according to the EHR performance reports, which would have been ideal; however, this was not feasible owing to limited resources and the time and costs associated with MRA. Being able to increase the sample sizes used in this analysis would have increased power and resulted in more precise estimates. It is unfortunate that the targeted initial sample size could not be reached owing to unforeseen barriers with conducting MRA within an additional health care system originally recruited for this study. Regardless, this remains the first and largest study, to our knowledge, to compare MRA, EHR, and misclassification-adjusted performance scores.

The data used in this study were from 21 primary care practices belonging to 1 large health care system using 1 EHR vendor participating in the H2O Project. Therefore, the results presented herein are not generalizable to all primary care practices and are likely to represent a best-case scenario for several reasons. First, participation in H2O was voluntary, and all participants had to be active users of EHR at the time of study enrollment. Hence, H2O participating practices may represent more progressive practices, that is, early adopters of new health information technology and value-based payment models. Second, the health care system used in this study may have had a more sophisticated EHR system, capable of capturing data more accurately than many EHR systems used by small, rural primary care practices. This would result in MRA and EHR sensitivities and specificities higher than what would be observed in many other primary care practices in Oklahoma and across the United States. Third, performance scores for the calendar year 2016 were examined for this project; therefore, all practices had received some level of the H2O practice improvement intervention by then. This too could have resulted in higher estimates of sensitivities, specificities, and performance scores than what might be the case in other practices in Oklahoma and the United States.

## Conclusions

The results of this study demonstrate that different data acquisition methods can result in different performance estimates. Given the importance of performance measurement to quality improvement efforts and value-based payment models, more extensive study is required to better understand the source of these differences so that improvements in care quality can be measured with confidence.

## Notes

Supplement. **eTable 1.** Prior Distributions Elicited From Subject Matter Experts for Medical Record Abstraction Sample Size Calculations **eTable 2.** Gibbs Specifications and Technical Details Used in the PropMisclassSampleSize Software for Aspirin, Blood Pressure, and Smoking Measure Sample Size Calculations **eTable 3.** Required Sample Sizes for the Aspirin Use Measure for Each of the 3 Criteria **eTable 4.** Required Sample Sizes for the Blood Pressure Control Measure for Each of the 3 Criteria **eTable 5.** Required Sample Sizes for the Smoking Cessation Counseling and Intervention Measure for Each of the 3 Criteria **eMethods 1.** Medical Record Abstraction Protocols and Extraction Tools **eMethods 2.** Methodology and Results for Medical Record Abstraction Sample Size Calculations **eMethods 3.** Bayesian Latent Class Model for 1 Population and 2 Imperfect Tests **eFigure 1.** Patient Flow Diagram for Determining Patients in Final Analysis by Aspirin Use Measure **eFigure 2.** Patient Flow Diagram for Determining Patients in Final Analysis by Blood Pressure Control Measure **eFigure 3.** Patient Flow Diagram for Determining Patients in Final Analysis by Smoking Cessation Counseling and Intervention Measure **eReferences.**
