# Supplementary material for: Validity of Medical Record Abstraction and Electronic Health Record–Generated Reports to Assess Performance on Cardiovascular Quality Measures in Primary Care
Source: JAMA Netw Open. 2020 Jul 28;3(7):e209411. doi: 10.1001/jamanetworkopen.2020.9411 (PMC7388024; doi:10.1001/jamanetworkopen.2020.9411)
Supplement: Supplement. — eTable 1. Prior Distributions Elicited From Subject Matter Experts for Medical Record Abstraction Sample Size Calculations eTable 2. Gibbs Specifications and Technical Details Used in the PropMisclassSampleSize Software for Aspirin, Blood Pressure, and Smoking Measure Sample Size Calculations eTable 3. Required Sample Sizes for the Aspirin Use Measure for Each of the 3 Criteria eTable 4. Required Sample Sizes for the Blood Pressure Control Measure for Each of the 3 Criteria eTable 5. Required Sample Sizes for the Smoking Cessation Counseling and Intervention Measure for Each of the 3 Criteria eMethods 1. Medical Record Abstraction Protocols and Extraction Tools eMethods 2. Methodology and Results for Medical Record Abstraction Sample Size Calculations eMethods 3. Bayesian Latent Class Model for 1 Population and 2 Imperfect Tests eFigure 1. Patient Flow Diagram for Determining Patients in Final Analysis by Aspirin Use Measure eFigure 2. Patient Flow Diagram for Determining Patients in Final Analysis by Blood Pressure Control Measure eFigure 3. Patient Flow Diagram for Determining Patients in Final Analysis by Smoking Cessation Counseling and Intervention Measure eReferences. [file jamanetwopen-3-e209411-s001.pdf]

Supplementary Online Content

Homco J, Carabin H, Nagykaldi Z, et al. Validity of medical record abstraction and electronic health record–generated reports to assess performance on cardiovascular quality measures in primary care. *JAMA Netw Open*. 2020;3(7):e209411. doi:10.1001/jamanetworkopen.2020.9411

eTable 1. Prior Distributions Elicited From Subject Matter Experts for Medical Record Abstraction Sample Size Calculations

eTable 2. Gibbs Specifications and Technical Details Used in the PropMisclassSampleSize Software for Aspirin, Blood Pressure, and Smoking Measure Sample Size Calculations

eTable 3. Required Sample Sizes for the Aspirin Use Measure for Each of the 3 Criteria

eTable 4. Required Sample Sizes for the Blood Pressure Control Measure for Each of the 3 Criteria

eTable 5. Required Sample Sizes for the Smoking Cessation Counseling and Intervention Measure for Each of the 3 Criteria

eMethods 1. Medical Record Abstraction Protocols and Extraction Tools

Methods 2. Methodology and Results for Medical Record Abstraction Sample Size Calculations

Methods 3. Bayesian Latent Class Model for 1 Population and 2 Imperfect Tests

eFigure 1. Patient Flow Diagram for Determining Patients in Final Analysis by Aspirin Use Measure

eFigure 2. Patient Flow Diagram for Determining Patients in Final Analysis by Blood Pressure Control Measure

eFigure 3. Patient Flow Diagram for Determining Patients in Final Analysis by Smoking Cessation Counseling and Intervention Measure

eReferences.

This supplementary material has been provided by the authors to give readers additional information about their work.

eTable 1. Prior Distributions Elicited From Subject Matter Experts for Medical Record Abstraction Sample Size Calculations

Parameter	Aspirin Use	Blood Pressure Control	Smoking Cessation Counseling and Intervention
Prevalence	Beta (13.59, 9.73)<br>N (0.58, 0.01)	Beta (14.71, 8.99)<br>N (0.62, 0.01)	Beta (11.16, 5.03)<br>N (0.69, 0.01)
Sensitivity	Beta (9.57, 5.18)<br>N (0.65, 0.01)	Beta (14.89, 1.46)<br>N (0.91, <0.01)	Beta (42.06, 2.77)<br>N (0.94, <0.01)
Specificity	Beta (417.49, 20.78)<br>N (0.95, <0.01)	Beta (28.29, 2.05)<br>N (0.93, <0.01)	Beta (43.76, 5.75)<br>N (0.88, <0.01)

N=Normal distribution

Estimates are presented as beta (α, β) and the equivalent normal (μ, σ^2) distributions.

eTable 2. Gibbs Specifications and Technical Details Used in the *PropMisclassSampleSize* Software for Aspirin, Blood Pressure, and Smoking Measure Sample Size Calculations

Number of Gibbs iterations to monitor:	10,000
Number of burn-in iterations:	4,000
Number of values sampled from preposterior:	4,000
Initial sample size:	1,000
Maximum feasible sample size:	100,000
Initial step:	250
March towards optimal sample size:	model-based

eTable 3. Required Sample Sizes for the Aspirin Use Measure for Each of the 3 Criteria

ACC (1 – α = 0.95)		ALC		MWOC (coverage=0.95)	
Length	Sample Size	Length	Sample Size	Length	Sample Size
0.1	> 100,000	0.1	> 100,000	0.1	>100,000
0.2	> 100,000	0.2	> 100,000	0.2	>100,000
0.3	8502	0.3	466	0.3	>100,000
0.4	0	0.4	0	0.4	0

*ACC=average coverage criterion; ALC=average length criterion; MWOC=modified worst outcome criterion.

©2020 Homco J et al. *JAMA Network Open*.

eTable 4. Required Sample Sizes for the Blood Pressure Control Measure for Each of the 3 Criteria

ACC (1 – α = 0.95)		ALC		MWOC (coverage=0.95)	
Length	Sample Size	Length	Sample Size	Length	Sample Size
0.1	> 100,000	0.1	> 100,000	0.1	>100,000
0.2	750	0.2	456	0.2	>100,000
0.25	82	0.25	76	0.25	637
0.3	26	0.3	26	0.3	56
0.4	0	0.4	0	0.4	0

*ACC=average coverage criterion; ALC=average length criterion; MWOC=modified worst outcome criterion.

eTable 5. Required Sample Sizes for the Smoking Cessation Counseling and Intervention Measure for Each of the 3 Criteria

ACC (1 – α = 0.95)		ALC		MWOC (coverage=0.95)	
Length	Sample Size	Length	Sample Size	Length	Sample Size
0.1	> 100,000	0.1	> 100,000	0.1	> 100,000
0.2	150	0.2	143	0.2	229
0.3	31	0.3	29	0.3	45
0.4	4	0.4	4	0.4	9

*ACC=average coverage criterion; ALC=average length criterion; MWOC=modified worst outcome criterion.

©2020 Homco J et al. *JAMA Network Open*.

eMethods 1. Medical Record Abstraction Protocols and Extraction Tools

The following are the chart abstraction instructions used for the Aspirin Use measure:

Part 1:

✓ **First, determine the Measurement Date (RepPeriod): January 1, 2016 – December 31, 2016**

We will be looking at a sample of patients diagnosed with ischemic vascular disease (IVD) according to EHR reports generated by the practice for the antithrombotic drug measurement review.

See your 'Chart Abstraction Template – Aspirin Use' Excel file for your list of sampled patients.

Part 2:

✓ **Now, let's determine if each patient chart meets our numerator criteria:**

1. Was there an active medication list entry for one of the listed antithrombotic drugs during the RepPeriod?

☐Yes – Meets criteria for Numerator 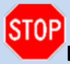

record as Numerator on spreadsheet

☐No – Continue with review to determine if exemption exists

Part 3:

✓ **Now, let's determine if each patient chart meet our exemption criteria:**

1. Does encounter indicate an adverse effect, allergy, or intolerance to aspirin **AND** clopidogrel (Plavix) medication?

©2020 Homco J et al. *JAMA Network Open*.

☐Yes – Meets medical reason on review STOP **record as meets Exemption criteria on spreadsheet**

☐No – Continue with review to determine why the measure is not met

2. Does the medication list or office note indicate another anticoagulant drug is prescribed?

☐Yes – Meets medical reason on review STOP **record as meets Exemption criteria on spreadsheet**

☐No – Continue with review to determine why the measure is not met

3. Does encounter note or problem list indicate a high risk of bleeding where the risks of therapy out weight the benefits (requires clinician determination)?

☐Yes – Meets medical reason on review STOP **record as meets Exemption criteria on spreadsheet**
Begin review of next patient chart

☐No – Continue with review to determine why the measure is not met

☐Unable to assess, clinician review not available – Continue with review to determine why the measure is not met

4. Does office note indicate the patient is receiving palliative care?

☐Yes – Meets exemption on review STOP **record as meets Exemption criteria on spreadsheet**

☐No – Continue with review to determine why the measure is not met

5. Is there documentation that a listed antiplatelet drug was recommended by a clinician but declined by the patient?

©2020 Homco J et al. *JAMA Network Open*.

☐Yes – Meets exemption on review 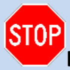

record as meets Exemption criteria on spreadsheet

☐No – Potential Quality Gap Identified

©2020 Homco J et al. *JAMA Network Open*.

The following are the chart abstraction instructions used for the Blood Pressure Control measure:

Part 1:

✓ **First, determine the Measurement Date (RepPeriod): January 1, 2016 – December 31, 2016**

We will be looking at a sample of patients diagnosed with hypertension age 18-85 years according to EHR reports generated by the practice for the controlling high blood pressure measurement review.

See your 'Chart Abstraction Template – Hypertension Controlling Blood Pressure' Excel file for your list of sampled patients.

Part 2:

✓ **Now, let's determine if each patient chart meets our numerator criteria:**

1. Look at the most recent office blood pressure recorded in a standard field in the EHR on or before the RepPeriod (must be within 1 year of the RepPeriod). Is the systolic blood pressure <140 and diastolic blood pressure <90?

☐Yes – Meets criteria for Numerator STOP **record as Numerator on spreadsheet**
☐No – Blood pressure is higher. Continue with review to determine if an exception (X) exists
☐No – Blood pressure is not measured. Continue with review to determine if an exception (X) exists

©2020 Homco J et al. *JAMA Network Open*.

Part 3:

✓ **Now, let's determine if each patient chart meets our exemption criteria:**

1. Is there a diagnosis code on the active problem list (on or before RepPeriod) or an office visit diagnosis code (on or within 1 year before RepPeriod) indicating pregnancy?

☐Yes – Meets medical reason on review STOP **record as meets Exemption criteria on spreadsheet**

☐No – Continue with review to determine if another exemption exists

2. Is there a diagnosis code on the active problem list (on or before RepPeriod) or an office visit diagnosis code (on or within 1 year before RepPeriod) indicating the patient has end stage renal disease (ESRD)?

☐Yes – Meets medical reason on review STOP **record as meets Exemption criteria on spreadsheet**

☐No – Continue with review to determine if another exception exists

3. Does the chart indicate that the patient is receiving palliative care?

☐Yes – Meets medical reason on review STOP **record as meets Exemption criteria on spreadsheet**

☐No – Possible quality gap (Stop)

©2020 Homco J et al. *JAMA Network Open*.

The following are the chart abstraction instructions used for the Smoking Cessation Counseling and Intervention measure:

Part 1:

✓ **First, determine the Measurement Date (RepPeriod): January 1, 2016 – December 31, 2016**

We will be looking at a sample of patients 18 years of age or older according to the EHR reports generated by the practice for the tobacco measurement review.

See your 'Chart Abstraction Template – Smoking Cessation and Intervention' Excel file for your list of sampled patients.

Part 2:

✓ **Now, let's determine if each patient chart meets our numerator criteria:**

1. Was there an assessment of tobacco use recorded in the EHR within the 24 months preceding the RepReriod?

☐Yes – Meets criteria for Numerator STOP **record as Numerator on spreadsheet**
☐No – Numerator not met, **Tobacco Status Not Assessed**, [Skip to Part 3]

2. Was the assessment that the patient is not a current tobacco user?

☐Yes – Meets criteria for Numerator STOP **record as Numerator on spreadsheet**
☐No – Continue to next question

©2020 Homco J et al. *JAMA Network Open*.

3. Was the assessment that the patient is a current tobacco user AND received a tobacco cessation intervention recorded in the EHR?

☐Yes – Meets criteria for Numerator STOP **record as Numerator on spreadsheet**

☐No – Continue to next question

4. Was the assessment that the patient is a current tobacco user AND a qualifying tobacco cessation medication was prescribed (discretely on medication list) within the 24 months preceding the RepPeriod?

☐Yes – Meets criteria for Numerator STOP **record as Numerator on spreadsheet**

☐No – Continue to Part 3

Part 3:

✓ **Now, let's determine if this chart meets our exemption criteria:**

1. Is there an adverse effect, allergy, or intolerance to a medication from the tobacco cessation medication list noted on or before the RepPeriod in the Allergy section?

☐Yes – Meets medical reason on review STOP **record as meets Exemption criteria on spreadsheet**

☐No – Continue with review to determine why the measure is not met

©2020 Homco J et al. *JAMA Network Open*.

2. Is there a diagnosis code on the active problem list (on or before RepPeriod) or an office visit diagnosis code (on or within 1 year before RepPeriod) indicating the patient is receiving palliative care*?

☐Yes – Meets medical reason on review **record as meets Exemption criteria on spreadsheet**

☐No – Stop patient does not meet measure

©2020 Homco J et al. *JAMA Network Open*.

The following data extraction tool was used to record chart abstraction results for the Aspirin Use measure:

PCP	IVD and ASA use		18 and older during measurement period	Measurement Period	IVD	Aspirin or Antithrombotic Medication	Exeptions: On Anticoagulant Allergy? High Risk for Bleeding? Etc	Numerator	Denominator	Total Den	Total Num	Total Rate
**Provider**	**Patient ID**	**DOB**	**Age**	**Encounter date**	**Diag**	**ASA/antiplatelet use**	**Exceptions**	**In Numerator**	**In Denominator**	**Total Denominator**	**Total Numerator**	**Total Rate**
										0	0	N/A

©2020 Homco J et al. *JAMA Network Open*.

The following data extraction tool was used to record chart abstraction results for the Blood Pressure Control measure:

PCP	Demographics		18-85 during *reporting period*	Must be in Reporting Period	Essential Hypertensio n Diagnosis	BP Reading <140/90	Exemptions Pregnancy ESRD Palliative Care	Denominator	Numerator	Total Den	Total Num	Total Rate
Provider	Patient ID	DOB	Age	Encounter date	(Y/N)	(Y/N)	Exemptions (Y/N)	In Denominator (Y/N)	In Numerator (Y/N)	Total Denominator	Total Numerator	Total Rate
										0	0	N/A

©2020 Homco J et al. *JAMA Network Open*.

The following data extraction tool was used to record chart abstraction results for the Smoking Cessation Counseling and Intervention measure:

PCP	Demographics		≥ 18 during *reporting period*	Must be in Reporting Period	Tobacco Assessed?	Non-Smoker?	Cessation Intervention Received?	Cessation Medication Prescribed?	Exemptions<br>Allergy<br>Palliative Care	Denominator	Numerator	Total Den	Total Num	Total Rate
Provider	Patient ID	DOB	Age	Encounter date	(Y/N)	(Y/N)	(Y/N)	(Y/N)	Exemptions (Y/N)	In Denominator (Y/N)	In Numerator (Y/N)	Total Denominator	Total Numerator	Total Rate
												0	0	N/A

©2020 Homco J et al. *JAMA Network Open*.

Methods 2. Methodology and Results for Medical Record Abstraction Sample Size Calculations

Prior information about Aspirin, BP, and Smoking performance scores and the sensitivity and specificity of EHR data to correctly classify patients as having performance met or not was elicited from two physicians whom are subject matter experts in performance measurement using the MATCH Tool.[1] The prior elicitation results, including the beta distributions and equivalent normal distributions are provided in Table 2.

The Bayesian sample size calculation methods described by Dendukuri et al. were used to determine the required sample sizes for chart abstraction (CA). The program *PropMisclassSampleSize* was used to calculate the sample sizes described here. This program uses R programs and WinBUGS scripts to estimate sample sizes for diagnostic accuracy studies assessing up to three imperfect tests. Prior information for the estimated disease prevalence and diagnostic test sensitivities and specificities are required for the program to estimate sample sizes. This software is publicly available for download and free of cost.[2]

Three different commonly used Bayesian sample size criteria were evaluated to better understand the range in sample sizes required based on both conservative and less conservative methods: Average Coverage Criterion (ACC), Average Length Criterion (ALC), and Modified Worst Outcome Criterion (MWOC). Each of these criterion uses a slightly different approach to determine the minimum sample size required to obtain the highest posterior density (HPD), i.e. the shortest credible interval on the posterior density, with a length of x that covers the posterior distribution of the parameter of interest with a given coverage probability, i.e. confidence level, of $(1 - \alpha)$.[3,4] At a minimum, credible interval lengths of 0.1, 0.2, 0.3, and 0.4 were evaluated for each Aspirin, BP, and Smoking measure sample size calculation. A coverage probability of 0.95 was used.

The ACC criterion holds the HPD credible interval length (x) constant while allowing the coverage probability $(1 - \alpha)$ to vary with the data.[2,3,5] For this analysis, 4,000 random data sets

©2020 Homco J et al. *JAMA Network Open*.

of test results were generated using the prior information outlined in Table 1. For each performance measure, a Bayesian latent class model was used to obtain the posterior distribution of the prevalence as well as the coverage of the HPD interval with a posterior credible interval length of *x*. For the Aspirin measure, the ACC criterion determined that the minimum sample size required to ensure that the average coverage across the 4,000 data sets was at least 95% with a fixed posterior credible interval length of 0.3 was 8,502 individuals.

The ALC criterion is similar to the ACC criterion. However, the coverage probability (1 – α) is held constant while allowing the HPD credible interval length (x) to vary with the data.[2,3,5] The ALC criterion ensures that the expected length of the posterior credible interval of fixed coverage is almost length *x* when averaged across all data sets. For the Aspirin measure, the ALC criterion determined that the minimum sample size required to ensure that the expected length of the posterior credible interval was 0.3 with a fixed posterior credible probability of 95% was 466 individuals.

The MWOC is most conservative criterion. MWOC ensures that at least 95% of the posterior credible intervals are no wider than the posterior credible interval length (x).[2,3,5] For the Aspirin measure, the MWOC criterion determined that the minimum sample size required to ensure that at least 95% of the predicted data sets have a HPD coverage of at least 95% for intervals of length at most 0.3 would require > 100,000 individuals.

The Gibbs specifications and technical details used in the *PropMisclassSampleSize* software across all methods are outlined in Table 3. For example, a sample size larger than 100,000 was considered infeasible. These are consistent with the default values provided in the *PropMisclassSampleSize* software. It has been suggested that these values generally provide reasonable sample size estimates.[2]

The sample size calculation results are described in Tables 4-6 separately for each performance measure.

Tables 4-6 report the required sample sizes estimates using the ACC, ALC and MWOC methods for Aspirin, BP, and Smoking. These results were shared and discussed with the Principal Investigator of the H2O Project. It was determined that resources were available to ensure the completion of 800 chart abstractions for all performance measures combined. Given this information and the results of the sample size calculations described above, it was determined that 500 chart abstractions would be conducted for Aspirin, 150 chart abstractions for BP, and 150 chart abstractions for Smoking. A simple random sample was used to select patients identified in each of the EHR-generated performance measures across the 28 participating practices. Therefore, patients were selected across both healthcare systems (EHR vendors) originally included in this project.

©2020 Homco J et al. *JAMA Network Open*.

Methods 3. Bayesian Latent Class Model for One Population and Two Imperfect Tests

```
model{

#likelihood

x[1:4] ~ dmulti(probability[1:4], n)
#x1 corresponds to Test 1 negative and Test 2 negative
#x2 corresponds to Test 1 negative and Test 2 positive
#x3 corresponds to Test 1 positive and Test 2 negative
#x4 corresponds to Test 1 positive and Test 2 positive

probability[1] <- prevalence*(1-Sensitivity1)*(1-Sensitivity2) + (1-
prevalence)*(Specificity1)*(Specificity2) #Test 1 negative and Test 2 negative
# p(D+)*p(T1-|D+)*p(T2-|D+) + p(D-)*p(T1-|D-)*p(T2-|D-)

probability[2] <- prevalence*(1-Sensitivity1)*(Sensitivity2) + (1-prevalence)*(Specificity1)*(1-
Specificity2) #Test 1 negative and Test 2 positive
# p(D+)*p(T1-|D+)*p(T2+|D+) + p(D-)*p(T1+|D+)*p(T2-|D+)

probability[3] <- prevalence*(Sensitivity1)*(1-Sensitivity2) + (1-prevalence)*(1-
Specificity1)*(Specificity2) #Test 1 positive and Test 2 negative
# p(D+)*p(T1+|D+)*p(T2-|D+) + p(D-)*p(T1+|D-)*p(T2+|D+)

probability[4] <- prevalence*(Sensitivity1)*(Sensitivity2) + (1-prevalence)*(1-Specificity1)*(1-
Specificity2) #Test 1 positive and Test 2 positive
# p(D+)*p(T1+|D+)*p(T2+|D+) + p(D-)*p(T1+|D-)*p(T2+|D-)

#Prior Set 2
prevalence ~ dunif(0.5,1)
#Test 1 CA
Sensitivity1 ~ dunif(0.5,1)
Specificity1 ~ dunif(0.5,1)
#Test 2 EHR
Sensitivity2 ~ dunif(0.5,1)
Specificity2 ~ dunif(0..5,1)
}
```

#posterior distributions for prevalence (i.e. performance score), sensitivity and specificity of Test 1, and sensitivity and specificity of Test 2 are generated based on combining the information provided in the likelihood and prior sections described above.

©2020 Homco J et al. *JAMA Network Open*.

eFigure 1. Patient Flow Diagram for Determining Patients in Final Analysis by Aspirin Use Measure

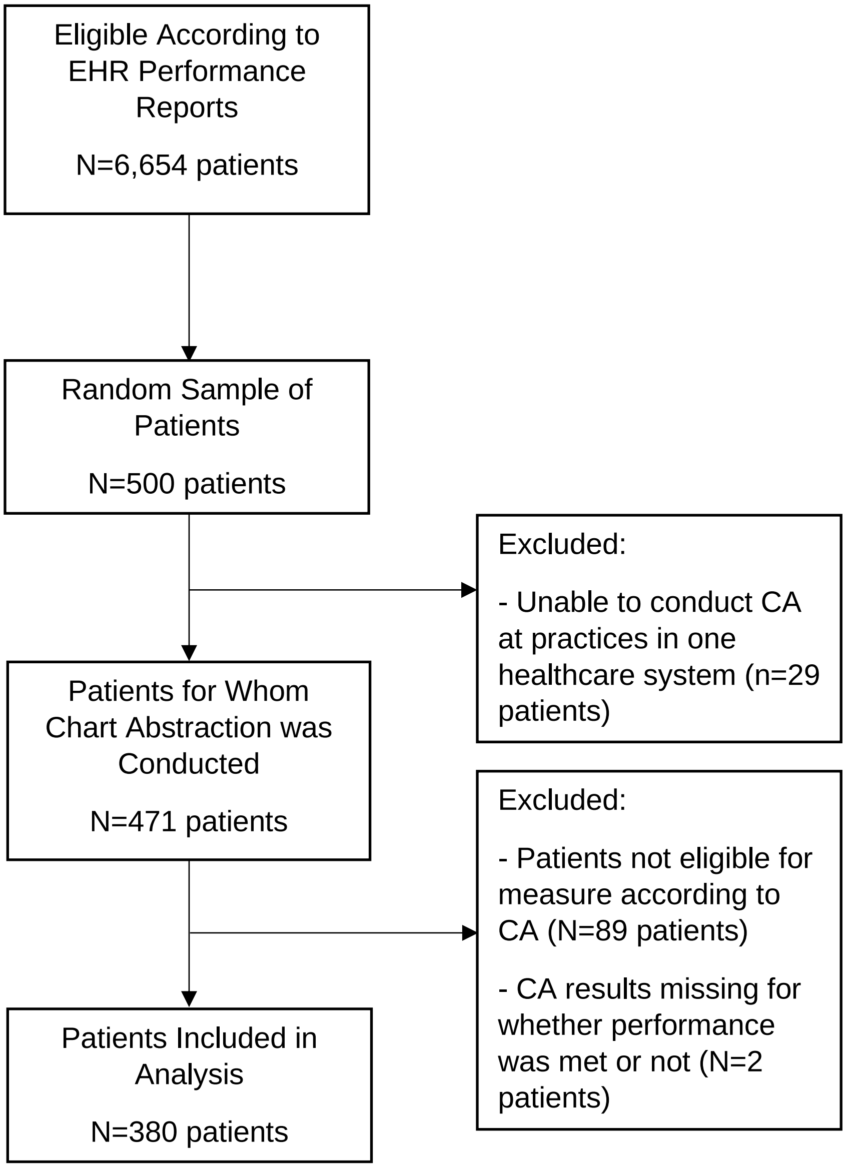

©2020 Homco J et al. *JAMA Network Open*.

eFigure 2. Patient Flow Diagram for Determining Patients in Final Analysis by Blood Pressure Control Measure

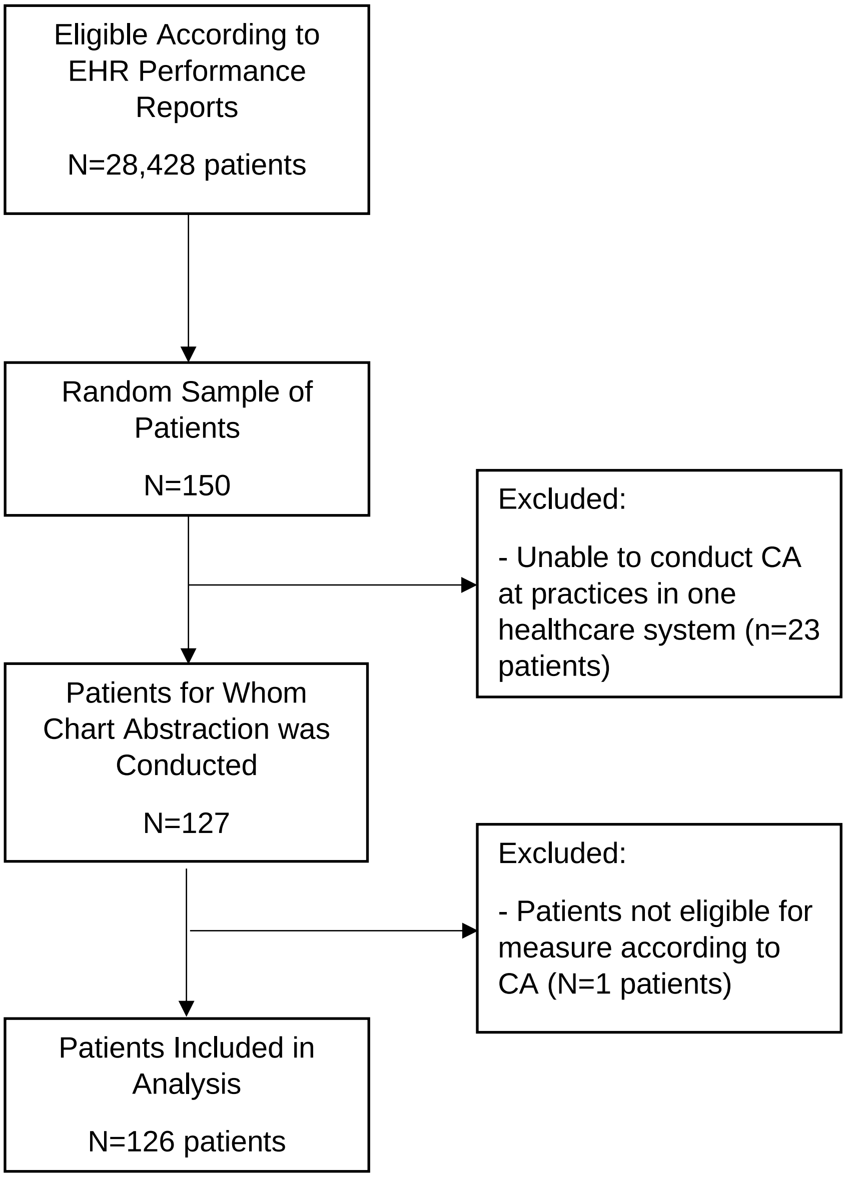

©2020 Homco J et al. *JAMA Network Open*.

eFigure 3. Patient Flow Diagram for Determining Patients in Final Analysis by Smoking Cessation Counseling and Intervention Measure

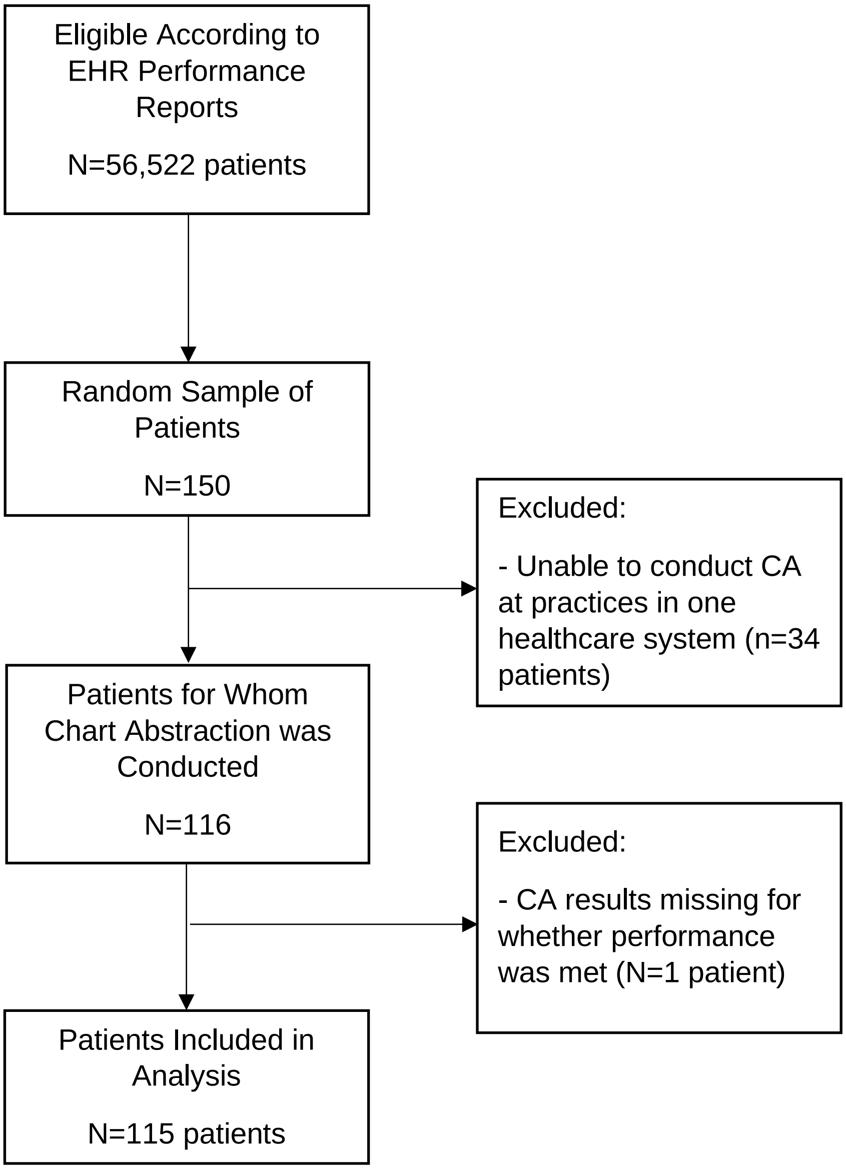

©2020 Homco J et al. *JAMA Network Open*.

eReferences.

1. Morris DE, Oakley JE, Crowe JA. A web-based tool for eliciting probability distributions from experts. *Environ Model Softw*. 2014;52:1-4.

2. Dendukuri N, Rahme E, Bélisle P, Joseph L. Bayesian sample size determination for prevalence and diagnostic test studies in the absence of a gold standard test. *Biometrics*. 2004;60(2):388-397.

3. Joseph L, du Berger R, Belisle P. Bayesian and mixed Bayesian/likelihood criteria for sample size determination. Stat Med. Apr 15 1997;16(7):769-781.

4. Collins J, Huynh M. Estimation of diagnostic test accuracy without full verification: a review of latent class methods. Stat Med. Oct 30 2014;33(24):4141-4169.

5. Cao J, Lee JJ, Alber S. Comparison of Bayesian Sample Size Criteria: ACC, ALC, and WOC. Journal of statistical planning and inference. 2009;139(12):4111-4122.

©2020 Homco J et al. *JAMA Network Open*.
